# Bloodletting as a Therapeutic Measure1Read before the Chemung County Medical Society, at Elmira, N. Y., May 18, 1897.

**Published:** 1897-10

**Authors:** C. A. Murray

**Affiliations:** Van Etten, N. Y.


### BLOODLETTING AS A THERAPEUTIC MEASURE.[1]

BY C. A. MURRAY, M. D., Van Etten, N. Y.

THE subject of bloodletting is probably at present the most unpopular theme known to the profession. It used to be the scapegoat upon whose head was heaped all our sins. It is that great mogul of which Prof. Reid has said : "Less slaughter I am convinced has been effected by the sword than by the lancet, that minute instrument of mighty mischief."

We need not be told that this is one of the most potent agents which the physician has to wield in the treatment of disease. It is

1. Read before the Chemung County Medical Society, at Elmira, N. Y., May 18, 1897.

potent for good and for evil. It may save life or it may destroy it. It is, therefore, obvious that the conscientious practitioner should be fully informed in regard to the effects and even remote consequences of venesection as a therapeutic agent. It is all the more necessary that this subject should be carefully investigated, as there is much reason to fear great mischief has been done in former years by its indiscriminate use.

The first obvious effect of bloodletting is the diminution of the actual quantity of blood circulating in the system. The second effect is impoverishing the quality of that blood; for immediately after a bleeding the blood vessels absorb watery fluid from every part of the system to compensate for their depleted condition and thus the blood is rendered more watery, less rich and less stimulating to the heart and arteries.

Another effect is to diminish the activity of the circulation. The vessels being less full, contract less powerfully upon their diminished contents and thus the pulse, that great index of the heart's action, becomes smaller. Less blood and of a less stimulating quality is sent to the capillaries and their action is diminished. The evidence of this is seen in the paleness which ensues after a bleeding and the same is true of every part of the system.

These, then, are the two effects produced on the vascular system by bloodletting—lessening the quantity and impoverishing the quality of the blood and as a consequence impairing the action of the heart and arteries. He, therefore, who would have his heart and brain fed with rich blood should avoid the use of the lancet. The energy of the brain depends upon the stimulus it receives from the arterial blood flowing through its substance. It is evident that whatever deranges the circulation must also derange the condition of the brain. Many physiologists claim that the actual quantity of blood in the brain is generally very much the same. It is mainly, therefore, the alteration in the composition of the blood that affects the brain. The condition of the circulation also affects it. For example, though the quantity of blood in the brain may not vary much, yet the quantity circulating through it in a given period may vary greatly, according to the frequency of the contractions of the heart; so, also, may the power or momentum with which the blood is driven by the heart to the brain, and in both these ways may the energy of the brain be impaired.

Now, as already stated, bloodletting impairs the action of the heart. The circulation becomes slower and the force with which it is driven to distant parts is lessened. The brain participates in this general effect. The blood is driven less forcibly to this organ and the quantity circulating through it in a given time is less. The result is, the energy of the brain is impaired. Accordingly giddiness, sickness at the stomach, and even fainting comes on. Also, many outward circumstances modify the effect of bloodletting, for instance, age; children, when bled to faintness, recover slowly, and this condition is not unattended with danger of convulsions and even death. Great caution, therefore, is necessary in bleeding children. From twenty to fifty years man is in his greatest vigor and it is during this period that the loss of blood can best be borne. In old age the action of the heart is impaired. The circulation becomes slower and the general powers fail, so that under the loss of blood reaction does not so readily take place, and if the quantity be large, great prostration will follow. Great caution here is also recommended.

Again, females, owing to the delicacy of their constitutions, do not bear the loss of blood as well as males. There are three epochs or periods in their lives which require attention in this relation—namely, menstruation, pregnancy and the period following the cessation of the menses. The influence of menstruation is so slight it need scarcely be noticed when considering the propriety of bloodletting. During pregnancy, however, loss of blood is better borne than in the natural condition and larger quantities are required to be abstracted to subdue disease. After the cessation of the menses, the natural tendency is to become plethoric. This is the natural consequence of the suspension of any physiologic discharge. Hence, at this period, diseases indicative of this condition are more common, such as apoplexy, paralysis, hemoptysis, scirrhus and many other maladies. Therefore venesection at this period is well borne.

Besides these constitutional peculiarities, others equally important result from habits and mode of life. A person bred to habits of industry and temperance, and, along with these, enjoying the benefit of wholesome food, pure country air and, above all, a mind free from care and depressing passions will bear venesection incomparably better than one whose constitution is undermined and whose vital energies are wasted by the unavoidable deprivations to which the poor are subject, or by the voluntary excesses, debauchery and intemperance of the rich. With regard to the use of the lancet in persons who have long been addicted to habits of intemperance great caution is necessary. In fact, I believe its use in such cases should be almost entirely interdicted. Dr. Watson (you will perceive we have to go back a good way to get authority on bloodletting) lays great stress on the buffy coat of the blood drawn, as indicating to some extent the height of the inflammation and hence the propriety of the bleeding. When the inflammation is high the blood drawn is frequently both buffed and cupped. Now I have shown that bloodletting reduces the quantity of the blood and impoverishes its quality and said, he who would have his heart and brain fed with rich blood should avoid the lancet. You will remember when the great Roman general, Regulus, was taken prisoner by the Carthagenians, he was offered the ransom of his life on what he considered dishonorable terms and when asked why he did not accept, he told them it was because he was a Roman, and he further said to those inferior Carthagenians. "if the bright blood which feeds my heart was like the slimy scum which stagnates in your veins, I should, doubtless, accept your ignoble terms and save my life." I quote this merely to show the value placed upon rich blood by all enlightened people.

But I come now to show you when this richness becomes excessive. When the blood becomes loaded with this inflammatory product, this buffy coat, this plasma, this organising matter, this coagulable lymph, which causes the pleura to adhere, the lungs to be filled with hepatisation, the trachea with fibrous false membrane, excessive inflammatory disease is present, and the surest, quickest and best way to reduce the blood to the standard of health is to use the lancet, which is the powerful means in our possession of reducing the pulse. I acknowledge hypodermatic injections of veratrum viride, morphia and other sedations may reduce the inflammation somewhat, yet these remedies obscure the symptoms and are not so rapid, so sure and reliable as bleeding.

Dr. Marshall Hall, many years ago, gave us a classification of diseases with reference to their ability to bear the loss of blood, which I have considered so able and so scientific that I have always followed it and have never had occasion to regret it. First, he classes congestion of the head and a tendency to apoplexy. These diseases, according to his classification, bear bloodletting best of all. Second comes inflammation of serous membranes, as of the pleura, peritoneum and the like. Then follows a long list of diseases not necessary to enumerate.

The first case to which I call your attention is one of puerperal eclampsia, which disease I believe to be a species of apoplexy and consequently coming under Dr. Marshall Hall's first classification, hence bearing bloodletting best of all.

CASE I.—Mrs. K., living near Spencer Village, was taken in convulsions after confinement, the second day. The attending physician called counsel with no beneficial result. The third day I was called alone to see the patient and found a case of profound coma, occasionally interrupted by a violent convulsion, accompanied by frothing at the mouth and opisthotonos. I found the pulse hard and full, which is generally characteristic of this disease. My resolve was instantly taken. I fortified myself by showing the utter hopelessness of the case in its present condition and proceeded to bleed in a recumbent posture, to actual syncope.

Fearing the bleeding had been excessive, I immediately gave her the benefit of position by allowing her head to hang off the bed, supported by an assistant, so that the blood might gravitate to the brain and thus support the heart by nervous influence, and not until a full half hour had elapsed was the volume of pulse sufficiently restored to allow the replacing of the head upon the pillow. In about an hour more she was seized with another convulsion. Thoroughly discouraged, I thought there was no hope and left the patient, promising to call again next morning. I failed to appreciate the fact that her brain had been under this terrible blood pressure two whole days and could not be expected to be immediately relieved. The next morning I was surprised to find the coma entirely gone, patient conscious, knew me, could talk, ultimately made a good recovery and I believe is alive today, though this happened about thirty years ago. I thought at the time the bleeding had been slightly excessive. I was, however, afterwards convinced that less bleeding would not have saved the patient. We must have courage to take sufficient blood to relieve the brain.

CASE II.—About ten years ago I was called early one morning to see Mrs. K., living about seven miles from Van Ettenville, and found her in convulsions at about the seventh month of utero gestation, in her first pregnancy. Dr. Dumond, of West Danby, was called as counsel. I suggested bleeding, according to my custom, to which he gladly gave assent, considering her case almost hopeless. She was accordingly bled about eighteen ounces, a large bleeding for a slender woman of eighteen years. This bleeding stopped the convulsions and relaxed the os uteri to such an extent that we succeeded in about eight hours in delivering her of a seven months' fetus, which lived about five hours. A remarkable symptom in this case was the total blindness which existed—a symptom which I believe commonly exists in eclampsia, but is frequently overlooked because the patient in her unconscious condition is unable to tell us she cannot see. We were thoroughly discouraged by this symptom, but kept her quiet and found next morning that her brain had cleared, sight was restored and that she was conscious. She made a good recovery. This case was considered the more remarkable from the fact that three other cases of this disease had happened in that vicinity which failed to be bled and all three died.

CASE III.—About five years ago I was called in the night to see Mrs. W., living about four miles from Van Ettenville, but on account of the darkness of the night and deep snow, I was unable to see her until daylight and then only by a circuitous route. I found her in the seventh month of pregnancy; primapara; had been in convulsions about eight hours. I immediately bled her largely, after which the convulsions were milder. I called Dr. Gee in counsel, who, like myself, could see no hope. I, however, remained with her and during the next night succeeded in delivering her of a dead fetus, but on account of the extreme prostration, dared make no attempt to accomplish the third stage of labor, which delayed for some hours. Unconsciousness continued. Yet this lady in her extremity insisted on getting well and did so. She made a good recovery and two years later was delivered of a healthy boy.

It may be interesting to note that this woman had another attack of eclampsia at this, her second confinement. After the labor was fully accomplished she had one terrible convulsion. But, profiting by my former experience with her, I bled her so quickly and freely that she didn't have another, but made a good recovery. A remarkable feature in this case was loss of voice. It was three days before she uttered the first articulate sound.

You will, therefore, perceive, while most severe cases of puerperal eclampsia die, I attribute the success I have had in the treatment of these cases to free bleeding. I believe there is no other rational treatment. Some physicians place great reliance upon hypodermic injections of veratrum viride, and justly so. Yet I believe this remedy does not clear the brain equal to bleeding and in large doses is equally debilitating and dangerous. Many practitioners claim its action is very uncertain and unreliable. Not that I would bleed in all cases of puerperal eclampsia or apoplexy. Far from it; I would never think of bleeding in such cases unless the pulse warranted it.

This leads me to speak of the pulse. To be a good judge of pulse requires great tact, experience and discrimination. Many kinds of pulse are described by authors for the guidance of students; more, perhaps, than are practically necessary. From this mass I

have culled a few which I think should be critically studied by every student who aspires to be even a fair judge of pulse, as follows: frequent or infrequent, quick or slow, strong or weak, full or small, hard or soft, regular or irregular, oppressed or free. The mere frequency of the pulse can be determined by a watch. All else, as you perceive, is the result of feeling and sensation. So, while seeing is believing, feeling is the naked truth. One can soon learn to discriminate between a quick pulse and a frequent pulse, and so of the others. The oppressed pulse is perhaps the most peculiar. This pulse at first seems weak and small, but becomes fuller and stronger by depletion. Its indications of disease, carefully studied, are very instructive.

To acquire this skill in judging of pulse we should educate ourselves, especially our fingers, cultivate that learned touch, that tactus eruditus, so that we may know to a certainty whether a given pulse will bear bleeding. This skill was possessed by our fathers in exquisite perfection, when bleeding was more in vogue than at present, and should bleeding continue to fall into disuse I think there is reason to fear we shall not qualify ourselves as thoroughly upon this point as they did.

You will perceive this knowledge is more necessary when I state the fact that two eminent professors were once found disputing sharply whether a certain pulse was hard or soft; such disputes ought not to occur among educated physicians. This judgment of pulse is equally necessary in cases which do not require bleeding, as it is an unerring guide to our treatment and tells us whether in a given case we ought to administer tonics and stimulants or sedatives and depressants.

The second classification of Dr. Marshall Hall, *i. e.*, bleeding in inflammation of serous membranes, reminds me of a circumstance which occurred when I was a student of medicine. My preceptor, Dr. C. C. Cook, of Newfield, was called to the adjoining town of Enfield to visit a patient that had been treated by a notorious quack of that town who called himself Dr. Woodruff. (We had no legal protection against quacks in those days.) He had treated this patient, as he thought, thoroughly and had given him large doses of his favorite cathartic, which he called "scratch about," but without result. Dr. Cook on examination found a case of acute peritonitis, abdomen distended and very tender. He placed his educated fingers upon the wrist and detected the kind of a pulse calling for the lancet. He immediately bled this patient to faintness; the moment he fainted the inflammatory spasm of the peritoneum was relaxed and the action of the bowels was terrible, the result, no doubt, of the large doses of "scratch about" he had taken and which the bleeding had set free. "There," said my preceptor, "Dr. Woodruff, clean up your patient, take good care of him and he will get well," which he did. This principle of relaxing inflammatory spasm of the peritoneum by bleeding to faintness was well understood at that time, was freely practised and was not then considered dangerous. The physician today who wields the lancet must have exquisite judgment regarding its use. We well know when blood is once drawn from the veins it cannot be replaced and the terrible prostration which follows may be fatal; hence prudent physicians hesitate and fear to take the responsibility. Therefore, while we deplete with one hand, we must be ready many times with the other to build up with powerful stimulants and tonics. Just why this is so, more than of old, the most scientific cannot tell; nevertheless the fact remains.

I will state here I have seen results equally remarkable from bleeding in cases of acute pleurisy, also in the pneumonias of forty-five years ago and which were saved by bleeding; by the treatment which would probably prove fatal in most cases at this time.

And now these facts are before us and the question arises, shall we entirely discard from our list of remedies this potent agent for controlling disease and thus probably sacrifice many valuable lives, or shall we educate ourselves, especially our fingers, to distinguish by the pulse, cases that require bleeding? I deplore the total decline of bloodletting. I believe the physician who, through fear, fails to bleed his patient when necessary, does not accomplish his whole duty. The fact that bloodletting in former years has been largely overdone is no excuse for its total suppression today. I am, therefore, one of the few who believe there are occasional cases, even in this age of debility and neuralgias, of nervous diseases and typhoid conditions, in which bleeding is not only beneficial, but absolutely necessary.

---

THE CARE OF CHILDREN'S TEETH.—The care of the teeth cannot be begun too early. If a child loses those of the first set prematurely the jaw contracts, there being nothing to prevent it from so doing; the second teeth have not space to stand properly and are crowded. Particles of food lodging between the teeth cause them to decay early. It is a wise precaution to teach a child to pass a thread of silk or dental floss between the teeth after eating, as well as to brush them regularly. Salt and water is a good antiseptic and answers for a dentifrice as well as many more elaborate preparations.—*Ladies' Home Journal.*

